# Evaluation of MRI Artifact in some selected centers in Kano Metropolis, Nigeria

**DOI:** 10.4314/ahs.v20i4.38

**Published:** 2020-12

**Authors:** Sidi Mohammed, Muhammad Abubakar

**Affiliations:** Bayero University, Department of Medical Radiography

**Keywords:** MRI artifacts, image quality, Kano metropolis

## Abstract

**Background:**

Magnetic Resonance Imaging (MRI) artifacts can occur due to hardware or software related problems, human physiologic phenomenon or physical restrictions. Careful study design and scanning protocols can prevent certain artifacts from occurring, but some are unavoidable.

**Study aims:**

The study aimed at evaluating MRI artifact in some selected centers in Kano metropolis, Nigeria.

**Methodology:**

A descriptive cross-sectional study was conducted involving both prospective and retrospective phases across three centres in the Kano metropolis from March 2019 to August 2019. Using the purposive sampling method, 3 centers were selected. A data capture sheet was designed for data collection.

**Results:**

Thirty five (50%) of the artifacts encountered were from the centreA, 28(40%) from the centre B, and 7(10%) from the centre C. Motion-induced artifact was the most frequently encountered artifact 26(37.1%), followed by wrap-around artifact 15(21.4%), and then frequency-induced artifact 13(18.6%). Thoracic spine MRI had the highest number of artifacts 28(40%), followed by brain 20(28.6%), and then lumbar spine 19(27.1%).

**Conclusion:**

In Kano metropolis the most encountered MRI artifact was the motion-induced artifact and thoracic spine MRI had the highest number of artifacts. The artifacts had a negative effect on image quality.

## Introduction

Magnetic resonance imaging is an imaging modality that uses a strong magnetic field and advanced computers to acquire images for the diagnosis of pathological conditions. It has two main advantages over other imaging modalities; it is not associated with ionizing radiation and provides excellent soft tissue contrast and characterization^1^. The modality is more sensitive to the molecular nature of tissues, and slight difference in tissue composition of normal gray and white matter of the brain. Therefore, it is the modality of choice for diseases involving the central nervous system, tumor staging, musculoskeletal disorders and congenital heart disorder^2^. However, the disadvantages of the modality include; long scan time, safety issues related to ferromagnetic materials within the patient, e.g. surgical clips, or electrical devices such as pacemakers^3^. The region of interest in the human body is usually placed near or surrounded by a coil, it uses a technique that stimulates the body to produce a radio-frequency (RF) signal, and it uses the receiver coil(as an antenna) to measure the signal. The signal is then processed using advanced computers to create the MRI image^4^.

Like any other type of diagnostic imaging, MRI is also susceptible to artifacts and these artifacts appear for a variety of reasons. Potential sources of the artifacts include non ideal hardware characteristics, intrinsic tissue properties and biological behavior, assumptions underlying the data acquisition and image reconstruction process, and poor choice of scanning parameters. Careful study design and scanning protocols can prevent certain artifacts from occurring, but some are unavoidable^5^. There are different types of artifacts, depending on their origin and can be classified into the following groups and each has its own causes, so also different strategies to minimize them. Truncation artifactis a type which occurs near sharp, high-contrast boundaries and are also known as the Gibbs phenomenon. It appears as multiple, alternating bright and dark lines “ringing”. They can be misinterpreted as a shrink in the spinal cord or a meniscal tear in the knee. Motion artifact caused by breathing, cardiac movement, CSF pulsation/blood flow, patient's movement, which creates ghost artifacts. It can be reduced by patient immobilization, cardiac/respiratory gating, saturation bands, or drugs that slow down the intestinal peristalsis. It can also reduce motion artifacts by using echo-planar imaging (EPI), a very fast MR imaging technique. Aliasing artifact occurs when the anatomical structures located outside the field of view are mapped at the opposite end of the image. It can eliminate them by increasing the field of view (FOV). Chemical shift artifact appears as dark or bright bands at the lipid-water interface and is seen especially in the case of fluid-filled structures surrounded by fat (e.g. eye balls in the orbits, bladder). It tends to be less prominent on T1-weighted images than on T2-weighted images. Interestingly, these artifacts have been used as a diagnostic aid, to confirm the presence of fat within lesions, e.g. in adrenal adenomas (Dual echo sequences/ out-of-phase image) or to accentuate the fat-water interfaces at visceral margins, thus helping in the evaluation of peripheral tumors for possible extra visceral extension^6^. But, in some cases it can be even more useful. 7

Artifacts affect the diagnostic quality, while others may be confused with pathology; this does not enable radiologists, radiographers and clinicians to provide diagnostic accuracy due to the artifacts created in the imaging modality. This account for problems in clinical decision making. In the standard practice IR images should be evaluated for artifacts, but empirical studies showed that MRI images are not evaluated for artifacts in some centers in Kano metropolis. The findings of the study are expected to serve as a baseline for making recommendations to relevant authorities, and serve as a guide to radiographers and radiologists. The study was aimed at evaluating MRI artifacts in some selected centers in Kano metropolis.

## Methods and materials

A descriptive cross-sectional study was conducted involving both prospective and retrospective phases across three centres; Centre A (0.3T Hitachi model of MRI scanner), Center B (0.2T GE, KC-2196 model of MRI scanner) and Centre C (1.5T Siemens, signal creator model of MRI scanner) in Kano metropolis, Nigeria from March 2019 to November 2019. There were 5 functional MRI scanners at the study, but only 3 agreed to participate in the study. An ethical approval to conduct the study was obtained from the Human Research and Ethics Committee of the Kano State Ministry of Health. The data was obtained from the procedures performed in the centers during the study period and the achieved. The data capture sheet was developed as an instrument of data collection instrument; the instrument was validated by an experienced senior colleague, the variables contained include; the name of the hospital, the type of the artifact encountered the region of the body where the variable was encountered and the effect of the artifact on the image quality. Each of the images was displayed on the monitor, evaluated by the researchers and the necessary information from the images was recorded on the data capture sheet. The frequency of the artifacts from the 3 centers was obtained using descriptive statistics.

## Results

Fifty percent of the evaluated MRI images were from centre A, 40% of centre B and the remaining 10% from centre C. Centre A had the highest number of encountered artifacts (35), followed by centre B (28) and centre C had the lowest (7). In centre A, thoracic spine had the highest number of artifact (23) followed by brain (9), in the same centre, cervical spine had the lowest number of artifact (1) followed by lumbar spine (2). In the centre, wrap-around artifact was the most frequent (14) followed by frequency artifact (11) and then motion induced artifact, the least frequent was the metal induced artifact. In centre B, lumbar had the highest number of artifact (11) followed by brain (9) and then thoracic spine (6). Cervical spine and knee joint had the lowest number of artifact (1) from each region. Lumbar spine had the highest (5) artifact in centre C and the brain had the lowest number of artifact (2). Motion induced artifact was the most frequent encountered in the centre, metal induced, susceptibility and Zebra were the least encounter artifacts (1) each.

In the 3 studied centres motion induced artifact was the most frequently encountered, thoracic and lumbar spine had the highest number (10) each, cervical spine was the least encountered (2) followed by brain (4). Wraparound artifact was the second most frequently encountered artifact (15) in the 3 centres; 14 out of the 15 were associated with the thoracic spine and the remaining 1 was associated with the knee joint. The third most frequently encountered artifact (13) in the three centres was frequency artifact; brain had the highest number (7) followed by lumbar spine (4). Zebra, chemical shift and susceptibility had the same frequency (4) each and were the fourth most frequently encountered artifacts in the 3 centres. Spike artifact was the least encountered artifact in the 3 centres (1) and associated with the brain, the second least encountered artifact in the 3 centres was the metal induced artifact (3). Two were associated with the brain while the remaining one was associated with lumbar spine. Thoracic spine was associated with highest number of artifact (28) followed by brain (20) and then lumbar spine (19). Knee joint had the lowest number of artifact (1) followed by cervical spine (2).

Thirteen out of the 26 motion induced artifact caused repeat of the MRI examination, the entire encountered (15) wrap-around artifact caused repeat, 8 out of the 13 frequency artifact caused repeat. Three out of the 4 Zebra artifact caused repeat of the MRI examination, 2 out of the 4 chemical shift artifact caused repeat, none of the susceptibility artifact causes repeat, 1 out of the 3 metal induced artifact caused repeat and the only one encountered spike artifact does not causes repeat of the MRI examination.

## Discussion

**Figure 1:**
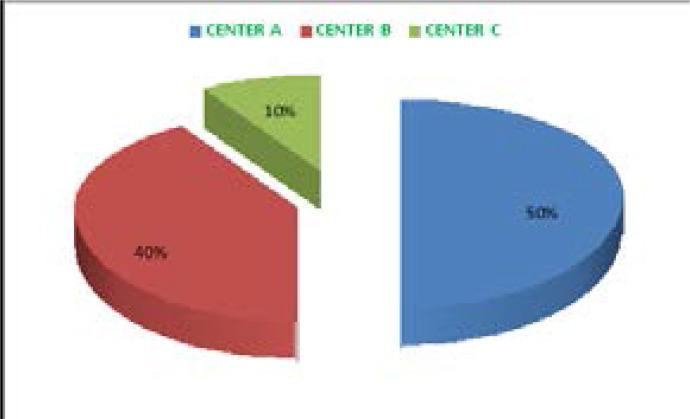
MR images from the study centres

The findings of the current study show that, 35 (50%) of the encountered artifacts were found in the series obtained from the Center A, 28 (40%) from the Center B, and 7 (10%) from the Center C as shown in Figure 1. The MRI scanner with a higher percentage of artifacts was 0.3T Hitachi model of the MRI scanner, owned by a private organization. The second MRI scanner was 1.5T Siemens, signal creator model of the MRI scanner, most recently installed MRI scanner in the study area and owned by the state government, the scanner with least encountered artifacts was 0.2T GE, KC-2196 model of MRI scanner both owned by another private organization. The findings of the study show that, the most frequently occurring artifacts in images acquired with 1.5T Siemens, signal creator model of MRI scanner and 0.2T GE, KC-2196 model of MRI scanner were motion-induced artifacts 13 (46.4%) and 4 (57.1%) respectively as shown in Tables 2 and 3. However, in 0.3T Hitachi model of MRI scanner motion induced artifact is third occurring artifact and constituted 9 (25.7%) as shown in Table 1.

**Table 2:** MRI series from Centre B

Body region	Types of artifacts								
	Motion induced artifact	Chemical shift artifact	Zebra artifact	Spike artifact	Frequency artifact	Susceptibility artifact	Metal induced artifact	Wrap around artifact	Total
**Brain**	02	00	02	03	00	01	01	00	09
**Cervical spine**	01	00	00	00	00	00	00	00	01
**Thoracic spine**	05	00	01	00	00	00	00	00	06
**Lumbar spine**	05	04	00	00	02	00	00	00	11
**Knee**	00	00	00	00	00	00	00	01	01
**Total**	13	04	03	03	02	01	01	01	28

**Table 3:** MRI series obtained from Center C

Body Region	Types of artifacts				
	Motion induced artifact	Metal induced artifact	Susceptibility artifact	Zebra Artifact	Total
**Brain**	00	01	01	00	02
**Lumbar** **spine**	04	00	00	01	05
**Total**	04	01	01	01	07

**Table 1:** MRI series from Centre A

Body region	Types of artifacts				
	Motion induced artifact	Wrap around artifact	Frequency artifact	Metal induced artifact	Total
**Brain**	02	00	07	00	09
**Cervical** **spine**	01	00	00	00	01
**Thoracic** **spine**	05	14	04	00	23
**Lumbar** **spine**	01	00	00	01	02
**Total**	09	14	11	01	35

Considering the total number of artifacts encountered in the three studied centers, motion-induced artifact is the most frequently encountered 26 (37.1%) as shown in Table 4. Motion-induced artifacts are more common with thoracic and lumbar MRI scan 10(14.3%) each as shown in Table 4; this is as a result of respiratory movement and gastrointestinal peristalsis. Furthermore, the findings of the study show that motion-induced artifact has a negative effect on the image quality. Fifty percent of the series associated with motion-induced artifacts caused repeat of the MRI examination as shown in Table 5. The repeat of the examination causes additional stress to the patient, reduces the departmental efficiency and increases the departmental running cost. The findings of the study are in agreement with the findings of the study conducted by^7^, which showed that, the most common types of artifacts were movement-induced artifacts (38 %). This may be due to the fact that, apart from voluntary movement, a lot of body organs are also associated with involuntary movement. In motion induced artifact the spin changes the frequency and therefore also phase compared to its original position. When the image is reconstructed, the position of the signal is put in the wrong place in the image. Random motion during the imaging sequence generally results in a blurring of the image, periodic motion produces ghost images. The correction strategies that can be used to eliminate or avoid such types of artifact include; breath holding, sedation, anesthesia, immobilization, signal averaging, breath triggering, and gradient moment nulling. The findings of the study also show that, the second most frequently occurring artifact is wrap- around artifact 15 (21.4%) as shown in Table 4. However, 14 (20%) was encountered in series acquired with 0.3T Hitachi model of the MRI scanner and were all associated with thoracic as shown in Table 1. The remaining 1 (1.4%) was encountered in series acquired with 1.5T Siemens, signal creator model of the MRI scanner and was associated with the knee joint as shown in table 2.

**Table 4:** Total number of MRI artifacts and the associated regions

Body Region	Types of artifacts								
	Motion induced artifact	Wrap around artifact	Frequency artifact	Zebra Artifact	Chemical shift artifact	Susceptibility artifact	Metal induced Artifac	Spike artifact	Total
**Brain**	04	00	07	02	00	04	02	01	20
**Cervical** **spine**	02	00	00	00	00	00	00	00	02
**Thoracic** **spine**	10	14	04	01	00	00	00	00	28
**Lumbar** **spine**	10	00	02	01	04	00	01	00	19
**Knee**	00	01	00	00	00	00	00	00	01
**Total**	26	15	13	04	04	04	03	01	70

**Table 5:** Effect of different type of artifact on image quality

Artifacts	Number of images that caused repeat	Number of images that do not caused repeat
**Motion induced**	13	13
**Wrap** **around**	15	0
**Frequency**	8	5
**Zebra**	3	1
**Chemical shift**	2	2
**Susceptibility**	0	4
**Metal induced**	1	2
**Spike**	0	1
**Total**	42	28

The findings of the study show that wrap-around artifact has a negative effect on the image quality. All the series associated with wrap-around artifacts caused repeat of the MRI examination as shown in Table 5. In wrap around artifact the part of the body outside the FOV will be wrapped-around into the image. In most cases this artifact is easily recognized and does not simulate disease; however, it can mask anatomical structures in the FOV. This type of artifact can be avoided by the use of the ‘No Wrap’ or ‘Double Matrix’ option switch, with a time penalty. The other solutions to wrap around artifact is to choose a larger field of view, adjust the position of the image center, or select an imaging coil which will not excite or detect spins from tissues outside of the desired field of view^8^.

Frequency artifact constituted 13 (18.6%) of the total artifacts encountered from the three centers as shown in table 4. However, 11 (15.7%) was encountered in images acquired with 0.3T Hitachi model of MRI scanner, 7 (10%) associated with brain MRI scan and 4 (5.7%) with thoracic as shown in Table 1. The remaining 2 (2.9%) was encountered in images acquired with 1.5T Siemens, signal creator model of the MRI scanner and was associated with lumbar spine as shown in Table 2. Frequency-induced artifact also has a negative effect on image quality because 8 (11.4%) caused repeat of the examinations as shown in Table 5. The frequency-induced artifacts are caused by ‘'dirty’ frequencies, faulty electronics, external transmitters, RF-cage leak, non-shielded equipment in the scanner room, metal in the patient, or when the door to the scanner room is left open can generate ‘'dirty’ frequencies. This usually requires an engineer to solve this kind of artifact, although, the door to the scanner room can be closed by non-qualified people as well. Furthermore, the findings of the study show that, Zebra artifact constituted 4 (5.7%) of the total artifacts encountered as shown in Table 4. However, 3 (4.3%) was encountered in series acquired with 1.5T Siemens, signal creator model of the MRI scanner and was associated with brain and thoracic spine as shown in Table 2. The remaining 1 (1.4%) was encountered in series acquired with 0.2T GE, KC-2196 model of the MRI scanner, and was associated with lumbar spine as shown in Table 3. The Zebra artifact has a negative effect on image quality because 3 (4.3%) caused repeat of the MRI examination. Basically, it appears as images with different phases from one side of the body to the other that alternatively add and cancel. These are especially seen in gradient echo techniques, interference patterns of superimposed pattern8. To avoid this problem you have to make sure that the patient is not touching the receive coil, or use No-Wrap option. The chemical shift artifact constituted 4 (5.7%) of the total artifacts encountered as shown in Table 4. It was encountered in series acquired with 1.5T Siemens scanner only as shown in Table 1, and was found to be associated with lumbar spine only. It also has a negative effect on image quality because 2 (2.9%) caused repeat of the examination as shown in Table 5. It is manifested as bright or dark outlines at fat-water interfaces. The chemical shift artifact can be corrected using am MRI device with a weaker magnetic field; use of the higher permeability receiver, selection of a larger matrix, phase TE or “spin-echo” (SE) sequences; larger gradient and special pulse sequences, such as fat saturation or inversion recovery and also decreasing the voxel size can be employed. The susceptibility artifact is seen in 4 (5.7%) of the series. However, 3 (4.3%) was encountered in series acquired with 1.5T Siemens scanner, 1 (1.4%) with 0.2T GE, KC-2196 model of the MRI scanner and both were associated with brain scan as shown in table 2 and 3. Fortunately, none causes repeat of the MRI examination as shown in Table 5, this show little effect on the image quality. It usually appears as light and dark spots with a spatial distortion in the neighboring anatomic structures.

The metal-induced artifact constituted 3 (4.3%) of the total series, 1 (1.4%) from each of the three scanners as shown in Tables 1, 2 and 3, two of the artifacts were associated with brain scan and one with lumbar spine as shown in Table 4. It has a minimal effect on image quality because only 1 (1.4%) caused a repeat of an examination. In metal-induced artifact geometric distortion of the image occurs which lead to the disappearance of the structures. These types of artifact can be corrected using techniques, such as flow compensation or cardiac triggering; to minimize or eliminate motion related artifacts. The removal of ferromagnetic foreign bodies; application of smaller voxel; shortening the response time; use of short echo time (TE); amplification of the receiver permeability; use of SE (especially fast SE) sequences and the MRI device with a weaker magnetic field are also some of the correction strategies^7^. Only 1 (1.4%) of the series was associated with spike artifact, it was obtained by 1.5T Siemens scanner and was associated with a brain scan. However, it has no negative effect on image quality because it didn't cause a repeat of the examination. In spike artifact, loss of single data points or data lines in the acquisition process due to spike forms external interfering signals or errors in the signal processing which can lead to a variable degree of artifact, sometimes showing a “crisscross” or “herringbone” pattern^9^.

## Conclusion

In Kano metropolis, MR images are associated with various types of artifacts; the most commonly encountered is the motion-induced artifact and thoracic spine is the body that was mostly affected. Furthermore, MRI artifacts have a negative effect on image quality.

## Figures and Tables

**Figure 2:**
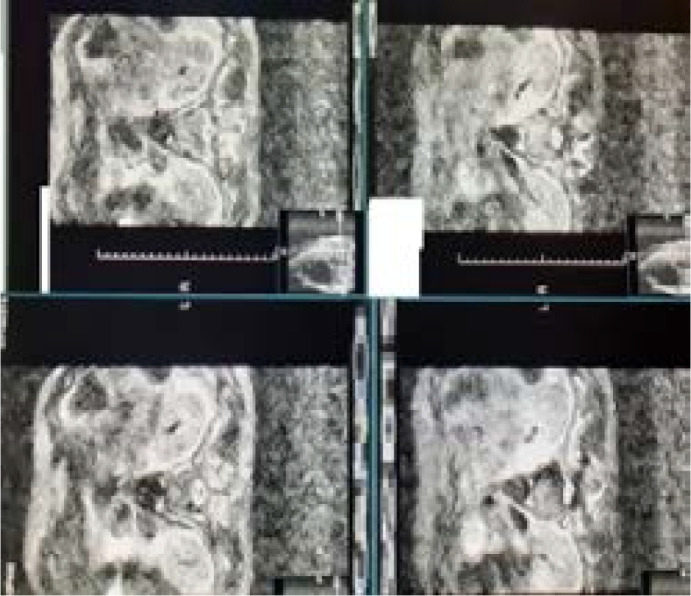
Motion induced artifact seen on lumbar spine MRI.

**Figure 3:**
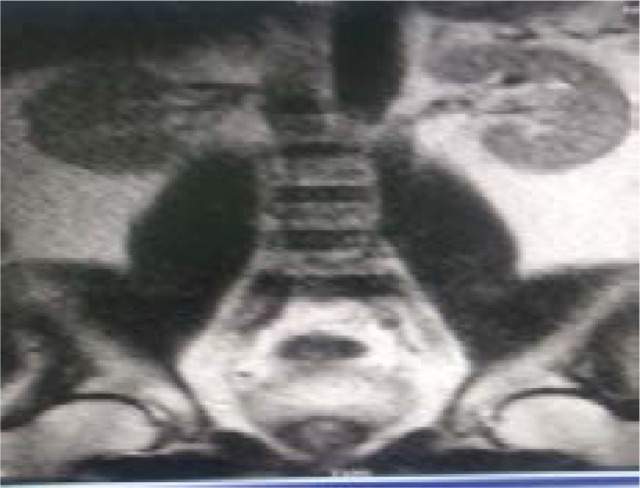
Chemical shift artifact seen on lumbar spine MRI

**Figure 4:**
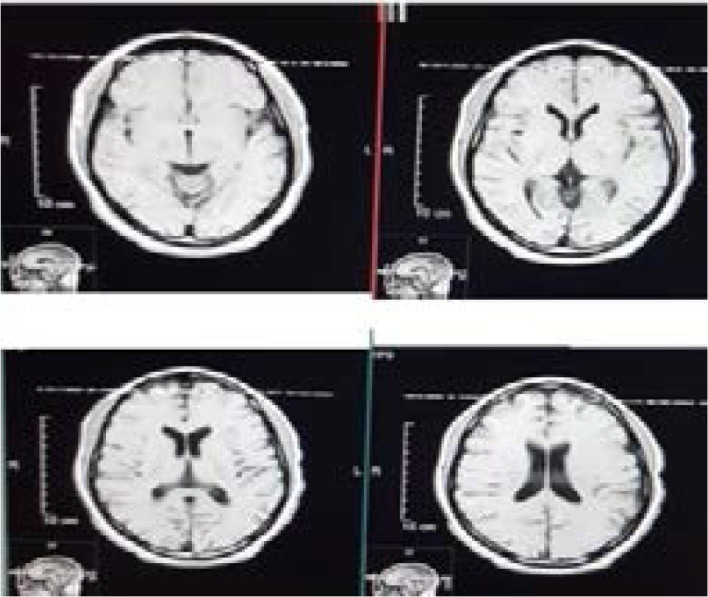
Frequency artifact as a result eye motion during acquisition of brain MRI.

**Figure 5:**
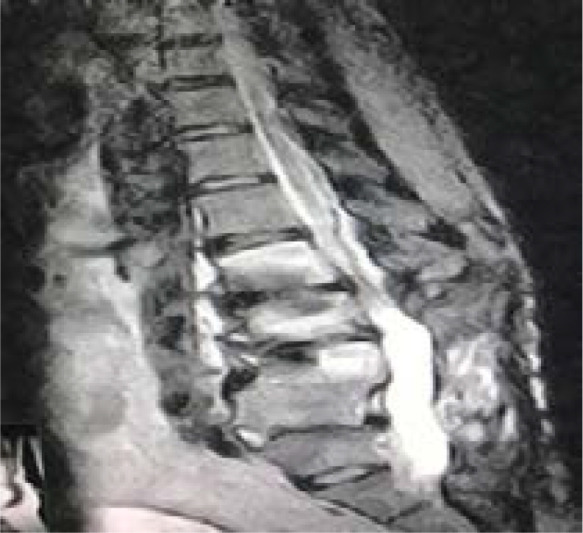
Metal induced artifact seen on lumbar spine MRI on patient with known inherent metallic implant

**Figure 6:**
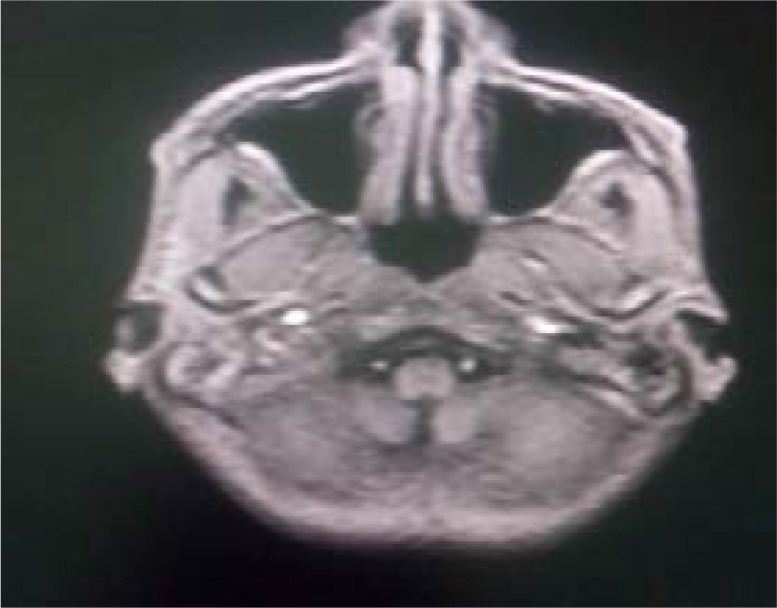
Spike artifact seen as diagonal line patterns on the image when observe carefully.

**Figure 7:**
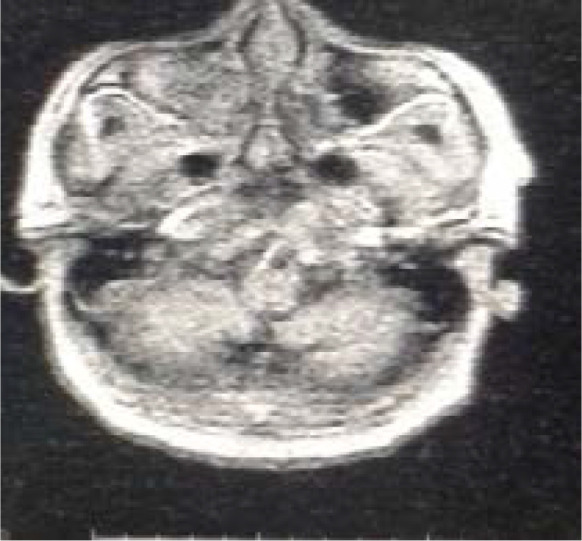
Susceptibility artifact seen on coronal brain MRI.
